# Data-processing routines for automated relaxation times and nuclear Overhauser effect measurements on a FT NMR spectrometer

**DOI:** 10.1155/S1463924681000047

**Published:** 1981

**Authors:** Jean-Paul Lemaire, Bernard Tiffon, Bernard Ancian

**Affiliations:** 1 Institut de Topologie et de Dynamique des Systemes de l'Université Paris VII associé au CNRS 1, rue Guy de la Brosse Paris 75005 France


### Data-processing routines for automated relaxation times and nuclear Overhauser effect measurements on a FT NMR spectrometer

**Jean-Paul Lemaire, Bernard Tiffon and Bernard Ancian**
*Institut de Topologie et de Dynamique des Systemes de l'Université Paris VII, associé au CNRS, 1, rue Guy de la Brosse, 75005 Paris, France.*

### Introduction

The value of measuring such nuclear magnetic resonance parameters as longitudinal and transverse relaxation times, and nuclear Overhauser effect enhancement factors has already been demonstrated [1]. While most modern Fourier Transform (FT) NMR spectrometers are fitted with the appropriate pulse sequence capabilities, they generally only provide a linear semi-logarithmic least-squares fit routine, which is insufficient for obtaining reliable relaxation time values [2-7]. Therefore, routines for a Jeol PFT 100 NMR spectrometer have been developed in this laboratory in order to compute on-line relaxation times using a two and three-parameter non-linear least-squares fit for most $T_1$ (IRFT, FIRFT, SRFT, Freeman-Hill modified IRFT) [8] and $T_2$ (CPMG) [9] measurement pulse sequences. A further routine increasing the reliability of NOE enhancement factor measurement [10, 11] from line areas in connection with phase correction has also been added. The basic principles involved and the algorithms of these routines are described here.

*Two parameter exponential least squares fit*

The problem consists of fitting a set of n points ($t_i$, $M_i$) by a two-parameter (Equilibrium magnetisation $M_0$ and relaxation time T) function involving a single exponential equation of one of the following types:

(a) Inversion recovery

$$M(t) = M_0 \left(1 - 2\exp\left(-\frac{t}{T}\right)\right)$$

(b) Fast inversion recovery

$$M(t) = M_0 \left(1 - \left(2 - \exp\left(-\frac{t_R}{T}\right)\right)\exp\left(-\frac{t}{T}\right)\right)$$

where $t_R$ is the repetition time of the pulse sequence.

(c) Freeman-Hill modified inversion-recovery

$$M(t) = 2M_0 \exp\left(-\frac{t}{T}\right)$$

(d) Saturation recovery

$$M(t) = M_0 \left(1 - \exp\left(-\frac{t}{T}\right)\right)$$

(e) $T_2$ measurement

$$M(t) = M_0 \exp\left(-\frac{t}{T}\right)$$

The error square sum is given by:

$$S = \sum_{i=1}^{n} [M_i - M(t_i)]^2$$

and must be minimised.

The five preceding types of function are particular cases of

$$M(t) = af(b,t) \text{ with } a = M_0 \text{ and } b = \frac{1}{T}$$

where f is a twice continuously derivable function.

If

$$S = \sum_{i=1}^{n} [M_i - af(b,t_i)]^2$$

is minimum, the first derivatives are equal to 0 :

$$\frac{\partial S}{\partial a} = -2 \sum_{i=1}^{n} [M_i - af(b,t_i)]\, f(b,t_i) = 0 \quad (1)$$

$$\frac{\partial S}{\partial b} = -2a \sum_{i=1}^{n} [M_i - af(b,t_i)]\, \frac{\partial f}{\partial b}(b,t_i) = 0 \quad (2)$$

Equation (1) leads to:

$$a = \frac{\sum_{i=1}^{n} M_i f(b,t_i)}{\sum_{i=1}^{n} [f(b,t_i)]^2} \quad (3)$$

Since the second derivative

$$\frac{\partial^2 S}{\partial a^2} = 2 \sum_{i=1}^{n} [f(b,t_i)]^2$$

is always positive, the value obtained by Equation (3) minimises S when b is known. Therefore we are faced with a one-parameter problem requiring minimisation of the quantity

$$S = \sum_{i=1}^{n} [M_i - g(b)\, f(b,t_i)]^2$$

where g (b) is a function defined by Equation (3). It can be shown that if a is positive, which is always the case here, the first derivative $\frac{dS}{db}$ has the same sign as:

$$E(b) = \sum_{i=1}^{n} A_iB_i \sum_{i=1}^{n} B_iC_i - \sum_{i=1}^{n} A_iC_i \sum_{i=1}^{n} B_i^2 \quad (4)$$

where $A_i = M_i$, $B_i = f(b,t_i)$ and $C_i = \frac{\partial f}{\partial b}(b,t_i)$

Since the function E(b) is simpler than $\frac{dS}{db}$ it thereby saves computation time. It can also be shown that if the n experimental points ($m_i$,$t_i$) are sufficiently close to a curve $M(t) = \alpha f(\beta,t)$ the value of S when E (b) = 0 (i.e. $\frac{dS}{db} = 0$)

is minimum of the function S. The function E (b) is monotonic near its zero value. This allows the use of a method of dichotomy over an interval $[b_1, b_2]$ which contains the optimal value. The following quantities $B_i$ and $C_i$ are calculated for $b_1$ and $b_2$ at each iteration for the relevant pulse sequence:

(a) Inversion recovery

$$B_i = 1 - 2\exp(-b\,t_i) \quad C_i = 2\,t_i \exp(-b\,t_i)$$

(b) Fast inversion recovery

$$B_i = 1 - (2 - \exp(-b t_R))\exp(-b\,t_i)$$

$$C_i = [\,2\,t_i - (t_i + t_R)\exp(-b\,t_R)\,]\exp(-b\,t_i)$$

(c) Freeman-Hill modified inversion recovery

$$B_i = 2\exp(-b\,t_i) \quad C_i = -2\,t_i \exp(-b\,t_i)$$

(d) Saturation recovery

$$B_i = 1 - \exp(-b\,t_i) \quad C_i = t_i \exp(-b\,t_i)$$

(e) $T_2$ measurement

$$B_i = \exp(-b\,t_i) \quad C_i = -t_i \exp(-b\,t_i)$$

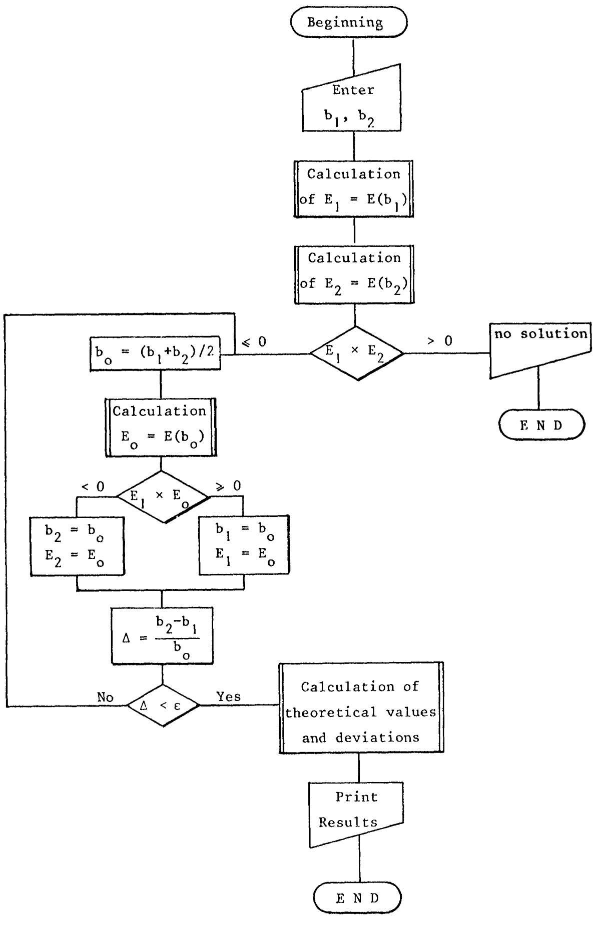

*Figure 1 Flow chart of the two-and three-parameter exponential least squares fit.*

If the optimum value of b is in the given search interval $[b_1, b_2]$ the signs of $E(b_1)$ and $E(b_2)$ are different (if not, an error message is printed and $b_1$ and/or $b_2$ must be changed). Then $E\left(\frac{b_1 + b_2}{2}\right)$ is computed and the search interval is reduced by half so that the signs of the values of E(b) at the two ends of the interval are different. At every iteration, the test of convergence compares the relative error $\left(\frac{b_2 - b_1}{b_2}\right)$ with the required precision $\epsilon$ (which is set equal to $10^{-4}$). When convergence occurs:

$$a = \frac{\sum_{i=1}^{n} A_i B_i}{\sum_{i=1}^{n} B_i^2}$$

and theoretical $M(t_i)$ values and deviations $[\,M(t_i) - M_i\,]$ are computed and results are printed as shown in the flow chart, Figure 1.

*Three-parameter exponential least-squares fit*

Some authors [4–6] have pointed out the need to add a constant term to the exponential function describing the magnetisation in $T_1$ measurements in order to take pulse imperfections into account. For $T_2$ measurements by the CPMG method, Hughes and Lindblom [12] use an expression of the so-called CPMG base-line which grows exponentially with a time-constant $T_2$; this is tantamount to adding a constant to the exponential decay of the echo amplitude. Therefore a three-parameter exponential fit for the following functions has been developed:

(a) Inversion-recovery

$$M(t) = -2\,M_0 \exp\left(-\frac{t}{T}\right) + C$$

(b) Fast inversion recovery

$$M(t) = -M_0\left[2 - \exp\left(-\frac{t_R}{T}\right)\right]\exp\left(-\frac{t}{T}\right) + C$$

(c) Saturation recovery

$$M(t) = -M_0 \exp\left(-\frac{t}{T}\right) + C$$

(d) $T_2$ measurement

$$M(t) = M_0 \exp\left(-\frac{t}{T}\right) + C$$

The four preceding functions are particular cases of

$$M(t) = a\,f(b,t) + C$$

where f is a twice continuously derivable function. If the error square sum S is minimum, the three first derivatives are equal to zero

$$S = \sum_{i=1}^{n} [M_i - a\,f(b,t_i) - C]^2$$

$$\frac{\partial S}{\partial a} = -2 \sum_{i=1}^{n} [M_i - a\,f(b,t_i) - C]\; f(b,t_i) = 0 \qquad (5)$$

$$\frac{\partial S}{\partial b} = -2a \sum_{i=1}^{n} [M_i - a\,f(b,t_i)\,] - C\,]\frac{\partial f}{\partial b}(b,t_i) = 0 \qquad (6)$$

$$\frac{\partial S}{\partial C} = -2 \sum_{i=1}^{n} [M_i - a\,f(b,t_i)\,] - C] = 0 \qquad (7)$$

*Figure 2 Listing of the two– and three–parameter fit. NPAR = number of parameters, PAR = array (3 x 2) used to store calculated parameters.*
*PAR (1, 1) = parameter a (heights calculation), PAR (2) = parameter a (areas calculation)*
*PAR (2, 1) = parameter T = $\frac{1}{b}$ (heights calculation),*
*PAR (2, 2) = parameter T (areas calculation)*
*PAR (3, 1) = parameter C (heights calculation) PAR (3, 2) = parameter C (areas calculation).*

*IFUNCT = 1 Inversion Recovery, = 2 Saturation Recovery, = 3 Fast Inversion Recovery, = 4 Freeman-Hill IR, = 5 $T_2$ Measurement.*
*TT = repetition time*
*IPR = 1 Intermediate calculations are printed, = 0 not printed NP number of points, TI, HG, AR = arrays (NP) which contain times, heights and areas.*
*$T_1$, $T_2$ = limits of the given interval of T*
*IK return code, = 0, no solution, = 1, solution with heights only, = 2, solution with areas only, = 3, solution with heights and areas.*

It follows from Equation (7) that:

$$C = \frac{\sum_{i=1}^{n} [M_i - a\, f(b,t_i)]}{n} \qquad (8)$$

Since the second derivative $\frac{\partial^2 S}{\partial C^2} = 2$ is always positive, the value of C obtained by Equation (8) minimises S when a and b are given. The problem is now a two-parameter minimisation.

$$S = \sum_{i=1}^{n} \left[M_i - a\, f(b,t_i) - \frac{\sum_{j=i}^{n} [M_j - a\, f(b,t_j)]}{n}\right]^2$$

If we set

$$A_i = M_i \qquad \bar{A}_i = A_i - \frac{1}{n}\sum_{j=1}^{n} A_j$$

$$B_i = f(b,t_i) \qquad \bar{B}_i = B_i - \frac{1}{n}\sum_{j=1}^{n} B_j$$

$$C_i = \frac{\partial f}{\partial b}(b,t_i) \qquad \bar{C}_i = C_i - \frac{1}{n}\sum_{j=1}^{n} C_j$$

we obtain

$$a = \frac{\sum_{i=1}^{n} A_i B_i - \frac{1}{n}\sum_{i=1}^{n} A_i \sum_{i=1}^{n} B_i}{\sum_{i=1}^{n} B_i^2 - \frac{1}{n}\left(\sum_{i=1}^{n} B_i\right)^2} = \frac{\sum_{i=1}^{n} \bar{A}_i \bar{B}_i}{\sum_{i=1}^{n} \bar{B}_i^2} \qquad (9)$$

Since $\frac{\partial^2 S}{\partial a^2}$ is always positive where b is given, the value of a obtained by (9) gives a minimum for S.

A one-parameter minimisation problem which can be treated as above, by replacing $A_i$, $B_i$, $C_i$ by $\bar{A}_i$, $\bar{B}_i$, $\bar{C}_i$ in Equation (4) is obtained. The algorithm and flow chart are the same as the previous ones (Figure 1). Depending on the pulse sequence used, the following quantities are calculated for $b_1$ and $b_2$ at each iteration:

(a) Inversion recovery

$$B_i = -2 \exp(-bt_i) \qquad C_i = 2 t_i \exp(-bt_i)$$

(b) Fast inversion-recovery

$$B_i = -[2 - \exp(-bt_R)] \exp(-bt_i)$$

$$C_i = [2 t_i - (t_i + t_R) \exp(-bt_R)] \exp(-bt_i)$$

(c) Saturation recovery

$$B_i = -\exp(-bt_i) \qquad C_i = t_i \exp(-bt_i)$$

(d) $T_2$ measurement

$$B_i = \exp(-bt_i) \qquad C_i = -t_i \exp(-bt_i)$$

Then $\bar{A}_i$, $\bar{B}_i$, $\bar{C}_i$, are calculated.

When convergence occurs

$$a = \frac{\sum_{i=1}^{n} \bar{A}_i \bar{B}_i}{\sum_{i=1}^{n} \bar{B}_i^2} \qquad C = \frac{1}{n}\left[\sum_{i=1}^{n} A_i - a \sum_{i=1}^{n} B_i\right]$$

and theoretical values and deviations are computed and results are printed.

The three-parameter as well as the two-parameter routine makes it possible to suppress any given experimental data. Line height and line area computation is sequential.

Both routines have been written in assembly language for a Jeol JEC 100 (Texas Instruments 980 A) computer. Computation is fast (a few seconds) although floating point operations are not hand wired and thus quite time-consuming. Computation time depends on the initial search interval which must contain the optimal value. Actually this is not a stringent condition since a rough value of the measured relaxation time is in any case required to set the proper measurement parameters (i.e. the $t_i$ values). The time-limiting factor is actually the speed of the printer on which reports are issued.

A single FORTRAN listing of the two- and three-parameter fits is presented in (Figure 2) so that it can be incorporated into the software of any FT NMR spectrometer computer equipped with a FORTRAN compiler and a sufficiently large core memory. A minimum knowledge of the basic NMR software (viz. the locations of stored $M_i$ and $t_i$ values) is, of course, also required.

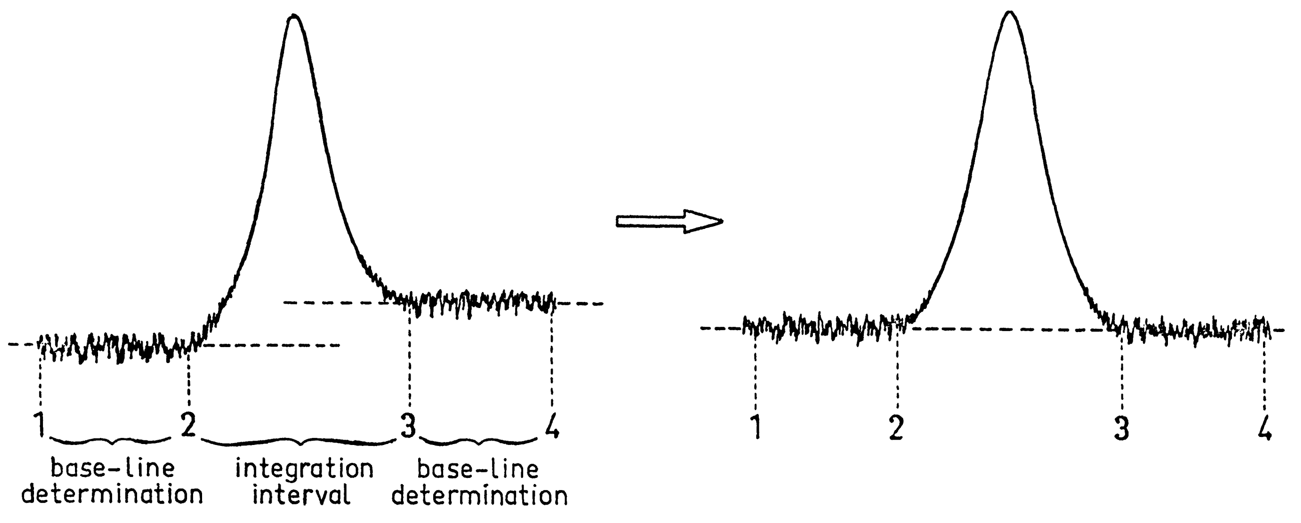

*Figure 3 Base-line level adjustment by phase correction. (The four points are chosen by the user).*

**Table 1. $^{13}C\ T_1$ measurements (in seconds) for $CS_2$ (20% $^{13}C$ enriched) byIRFT at 25 MHz (at room temperature).**

| Semi-log plot | Two-parameter fit | | Three-parameter fit | |
|---|---|---|---|---|
| Heights | Heights | Areas | Heights | Areas |
| 40.3 | 43.6 | 43.2 | 42.5 | 41.0 |
| 40.5 | 43.8 | 43.4 | 43.1 | 41.1 |
| 40.4 | 42.7 | 42.8 | 48.5 | 47.4 |
| 39.1 | 43.4 | 42.7 | 44.4 | 44.6 |
| 42.0 | 44.1 | 44.1 | 49.3 | 49.2 |

Table 1 compares $^{13}C\ T_1$ values obtained by the linear semi-logarithmic plot and by the two- and three-parameter exponential fits. Although some authors claim that a three-parameter exponential fit gives better results [4-6] for $T_1$ measurements, it can be seen here that the two-parameter fit gives the most consistent results for line heights and for line areas. Nevertheless, for $T_2$ CPMG measurements, a three-parameter exponential fit is needed (unpublished results) because of the CPMG base line [12]. For $T_2$ measurement, only line height should be considered, due to the inhomogeneity of the magnetic field over the sample volume.

### *NOE calculation from line areas: base-line adjustment by phase correction*

The NOE enhancement factor $\eta$ is defined as

$$\eta = (M_e - M_o)/M_o$$

where $M_e$ is the signal intensity of the observed spins (i.e. $^{13}C$) when interacting spins (i.e. $^1H$) are irradiated, and $M_o$ is their intensity when they are not [1, 10, 11]. As a difference between two NMR signal intensities is involved, an accurate measurement of line heights or line areas is required. If line areas are used (for example in the case of long accumulation), the integration base-line level is critical, especially for broad lines. Moreover, line areas are highly dependent upon phase correction, i.e. slight phase error can induce large uncertainty on line area. Therefore, the routine developed is one in which the average value of two mean noise levels on each side of a given line is used to compute the line area (Figure 3) by adjusting the two phase correction parameters until the two base-line levels are equal (i.e. perfect phase correction). This procedure requires non-overlapping NMR lines; however, should NMR lines overlap, the NOE enhancement factor can only be computed from the line heights. Incorporating this routine into a FT NMR computer software is quite complicated and requires a very good knowledge of the original software. Further details about this routine can be provided upon request. Measurement of the NOE enhancement factor for the carbonyl group of acetone shows (Table 2) that line areas with base-line adjustment give the most consistent values.

**Table 2. $^{13}C$-($^1H$) NOE enhancement factor measurement for the carbonyl group of acetone (33,33% v/v in n-pentane) at 25 MHz (50 scans, spectral width 1 KHz, 4 K data points in the frequency domain, 40 points integration).**

| | Line height | Line area |
|---|---|---|
| without | 0.149 | 0.063 |
| base-line | 0.160 | 0.130 |
| adjustment | 0.264 | 0.142 |
| | 0.048 | 0.073 |
| with | 0.189 | 0.073 |
| base-line | 0.218 | 0.087 |
| adjustment | 0.223 | 0.083 |
| | 0.051 | 0.086 |

REFERENCES

[1] Farrar T.C. and Becker E.D., "Pulse and Fourier Transform NMR", 1971, Academic Press, New York.

[2] De Fontaine D.L., Ross D.K. and Ternai B., *Journal of Magnetic Resonance,* 1975, 18, 276.

[3] Gerhards R. and Dietrich W., *Journal of Magnetic Resonance,* 1976, 23, 21.

[4] Leipert T.K. and Marquardt D.W., *Journal of Magnetic Resonance,* 1976, 24, 181.

[5] Sass M. and Ziessow D., *Journal of Magnetic Resonance,* 1977, 25, 263.

[6] Kowalewski J., Levy G.C., Johnson L.F. and Palmer L., *Journal of Magnetic Resonance,* 1977, 26, 533.

[7] Brunetti A.H., *Journal of Magnetic Resonance,* 1977, 28, 289.

[8] Levy G.C. and Peat I.R., *Journal of Magnetic Resonance,* 1975, 18, 500.

[9] Vold R.L., Vold R.R. and Simon H.E., *Journal of Magnetic Resonance,* 1973, 11, 283.

[10] Harris R.K. and Newman R.H., *Journal of Magnetic Resonance,* 1976, 24, 449.

[11] Canet D., *Journal of Magnetic Resonance,* 1976, 23, 361.

[12] Hughes D.G. and Lindblom G., *Journal of Magnetic Resonance,* 1977, 26, 469.

ACKNOWLEDGEMENT

The authors wish to thank Jeol Ltd, Tokyo, and Jeol (Europe) S.A., Paris, for their contribution to this work.

